# Microfluidic Fabrication of Click Chemistry-Mediated Hyaluronic Acid Microgels: A Bottom-Up Material Guide to Tailor a Microgel’s Physicochemical and Mechanical Properties

**DOI:** 10.3390/polym12081760

**Published:** 2020-08-06

**Authors:** Thomas Heida, Oliver Otto, Doreen Biedenweg, Nicolas Hauck, Julian Thiele

**Affiliations:** 1 Institute of Physical Chemistry and Polymer Physics, Leibniz-Institut für Polymerforschung Dresden e. V., 01069 Dresden, Germany; heida@ipfdd.de (T.H.); hauck@ipfdd.de (N.H.); 2 Center for Innovation Competence: Humoral Immune Reactions in Cardiovascular Disorders, University of Greifswald, Fleischmannstr. 42, 17489 Greifswald, Germany; oliver.otto@uni-greifswald.de; 3 German Center for Cardiovascular Research e. V., University Medicine Greifswald, Fleischmannstr. 42, 17489 Greifswald, Germany; 4 Clinic for Internal Medicine B, University Medicine Greifswald, Fleischmannstr. 8, 17475 Greifswald, Germany; doreen.biedenweg@uni-greifswald.de

**Keywords:** hyaluronic acid microgels, bio-orthogonal click chemistry, droplet microfluidics, trifunctionality

## Abstract

The demand for tailored, micrometer-scaled biomaterials in cell biology and (cell-free) biotechnology has led to the development of tunable microgel systems based on natural polymers, such as hyaluronic acid (HA). To precisely tailor their physicochemical and mechanical properties and thus to address the need for well-defined microgel systems, in this study, a bottom-up material guide is presented that highlights the synergy between highly selective bio-orthogonal click chemistry strategies and the versatility of a droplet microfluidics (MF)-assisted microgel design. By employing MF, microgels based on modified HA-derivates and homobifunctional poly(ethylene glycol) (PEG)-crosslinkers are prepared via three different types of click reaction: Diels–Alder [4 + 2] cycloaddition, strain-promoted azide-alkyne cycloaddition (SPAAC), and UV-initiated thiol–ene reaction. First, chemical modification strategies of HA are screened in-depth. Beyond the microfluidic processing of HA-derivates yielding monodisperse microgels, in an analytical study, we show that their physicochemical and mechanical properties—e.g., permeability, (thermo)stability, and elasticity—can be systematically adapted with respect to the type of click reaction and PEG-crosslinker concentration. In addition, we highlight the versatility of our HA-microgel design by preparing non-spherical microgels and introduce, for the first time, a selective, hetero-trifunctional HA-based microgel system with multiple binding sites. As a result, a holistic material guide is provided to tailor fundamental properties of HA-microgels for their potential application in cell biology and (cell-free) biotechnology.

## 1. Introduction

Microgels are solvent-swollen macromolecular networks forming finite structures ranging in size from tens of micrometers to the nanometer scale [1]. Due to their unique versatility regarding the adjustment of mechanical and physicochemical properties, microgels have attracted attention as promising substrates, e.g., for engineered extracellular matrices (ECMs) [2,3] drug delivery systems [4,5,6], or cell-free biosynthesis environments [7,8,9]. Compared to bulk gels, the higher surface-to-volume ratio of microgel particles results in a remarkably enhanced molecular mass transport between the polymer network and the surrounding (micro-)environment [10,11]. Moreover, microgels of different size, architecture, or chemical features can be mixed, hierarchically assembled, or embedded into a polymer matrix forming supragels and multiphasic hydrogels, respectively [12]. For instance, in the field of tissue engineering, these composites reassemble the complex heterogeneity of the ECM more closely than macroscopic bulk hydrogels [13]. However, when applying well-established emulsion polymerization techniques for microgel formation—e.g., membrane emulsification, spray drying, or precipitation polymerization—products with rather limited control over particle uniformity, morphology, and functionality are obtained [14]. Droplet microfluidics (MF), as the method of choice, overcomes these limitations in design flexibility, allowing for precisely tailoring microgel particles regarding—among other parameters—size, shape, permeability, elasticity, and functionality. This distinct control originates from the ability to precisely manipulate flow rates, flow pattern formation, and mixing of microgel precursors inside microfluidic devices, which are commonly fabricated by combined photo and soft lithography [15,16]. Due to short dwell times and fast mixing dynamics facilitated by customized microchannels, even fast-gelling polymer materials can be processed without forming spatially heterogeneous polymer networks [17]. To take advantage of microfluidic precision, prior to droplet templating, the selection of polymer precursor material(s) and corresponding crosslinking strategy, respectively, is crucial in addressing the desired material characteristics. In particular in biomaterial design, besides synthetic polymers—e.g., poly(ethylene glycol) (PEG), poly(*N*-isopropyl acrylamide) (PNIPAM), or poly(lactic-*co*-glycolic acid) (PLGA)—natural polysaccharides, such as agarose, alginate, heparin, or hyaluronic acid (HA) have received broad attraction. These naturally occurring polymers are non-toxic, non-immunogenic, biocompatible, and mostly biodegradable [11,18]. In particular, HA, playing an essential role in cell proliferation and migration as a component of the ECM [19,20], has been widely applied due to its broad versatility in terms of chemical modification strategies [21]. For instance, HA-based microgels (HA-microgels) have been modified with cell-binding sites, such as fibrinogen or the tripeptide arginine-glycine-aspartic acid (RGD)-sequence, to create customized microenvironments for mimicking the native ECM [22,23]. Beyond matrix engineering, HA-microgels have been employed as delivery systems for therapeutic proteins, such as Herceptin [24], or implemented as microbioreactors to perform cell-free protein synthesis in tailored microenvironments [7,8,25]. To address the challenges of engineering tailored biomaterials, and at the same time, avoiding undesirable reactions between the crosslinking chemistry and (bio-)components that are entrapped during microgel formation or attached to the polymer precursor(s), the construction of biocompatible microgel networks is closely associated with the use of click chemistry strategies. Defined by Sharpless and coworkers, chemical conjugation via click chemistry is set by certain criteria: “wide in scope, give very high yields, generate only inoffensive byproducts that can be removed by nonchromatographic methods, and be stereospecific (but not necessarily enantioselective)” [26]. However, to realize microgel-based environments of broad diversity and complexity, the choice of crosslinking strategy and corresponding microgel design are critical to adapt structure, (physico-)chemistry, and mechanics for highly specific applications.

To this end, we highlight the potential of click chemistry-mediated HA-microgel formation via droplet microfluidics by presenting a holistic material guide on the microfluidic design and characterization of HA-microgels based on three independent and highly selective bio-orthogonal click reactions: Diels–Alder [4 + 2] cycloaddition, SPAAC, and UV-initiated thiol–ene reaction. To enable straight-forward network formation utilizing homobifunctional PEGs as crosslinkers, in all three approaches, the chemical modification of HA-precursor materials is screened in-depth prior to their microfluidic processing into uniform water-in-oil (W/O) emulsions. By introducing a tailored microchannel design, we ensure efficient mixing of even fast-gelling polymer precursors and prepare microgels, whose swelling properties, permeability, (thermo)stability, and elasticity are investigated in detail. Furthermore, we demonstrate the overall flexibility of a MF-based HA-microgel design by preparing non-spherical microgels and introduce a hetero-trifunctional HA-microgel for selective and multiple attachments, e.g., of biomolecules. With this holistic material selection and processing guide at hand covering a broad spectrum of microgel key properties, we hope to contribute to the ongoing development of well-defined microgel systems, mainly based on hyaluronic acid, in the field of biosciences.

## 2. Materials and Methods

### 2.1. Materials and Instrumentation

Unless otherwise stated, all chemicals were used as received. Sodium hyaluronate (41–65 kDa) was purchased from Lifecore Biomedical (Chaska, MN, USA). PEG-maleimide_2_ (5000 g mol^−1^), PEG-norbornene_2_ (6000 g mol^−1^), PEG-azide_2_ (5000 g mol^−1^), and biotin-PEG-maleimide (5000 g mol^−1^) were purchased from Creative PEG Works (Chapel Hill, NC, USA). Fluorescein isothiocyanate-dextrans (4, 40, 150, 250, 500, 2000 kDa), deuterium oxide (D_2_O), *N*-hydroxysuccinimide (NHS), dibenzocyclooctyne-amine (DBCO-amine), Span^®^ 80, dibenzocyclooctyne-PEG_4_-maleimide (DBCO-PEG_4_-mal), 2-hydroxy-4-(2-hydroxyethoxy)-2-methyl-propiophenone (Irgacure^®^ 2959), and Grace Bio-Labs SecureSeal™ imaging spacer were obtained from Sigma-Aldrich (St. Louis, MO, USA). Dibenzocyclooctyne-sulfo-PEG_4_-amine (DBCO-sulfo-PEG_4_-amine) and tris-(2-carboxyethyl) phosphine hydrochloride salt (TCEP-HCl) were purchased from Iris Biotech GmbH (Marktredwitz, Germany). 4-(4,6-dimethoxy−1,3,5-triazin-2-yl)-4-methylmorpholinium chloride (DMTMM), lithium phenyl(2,4,6-trimethylbenzoyl) phosphinate (LAP), and 5-methylfurfurylamine were purchased from TCI (Portland, OR, USA). Atto425-streptavidin, Atto565-maleimide, Atto565-azide, and Atto647 N-maleimide were purchased from ATTO-TEC GmbH (Siegen, Germany). 1-Ethyl-3-(3-dimethylaminopropyl) carbodiimid-hydrochlorid (EDC-HCl) was purchased from Carl Roth GmbH (Karlsruhe, Germany). HFE-7500 (3 M™) was purchased from IoliTec (Heilbronn, Germany). (Tridecafluoro−1,1,2,2-tetrahydrooctyl) trichlorosilane was obtained from Gelest (Morrisville, PA, USA). 3-(2-pyridyldithio) propionyl hydrazide (PDPH) was purchased from CovaChem (Morrisville, PA, USA). 1*H*,1*H*,2*H*,2*H*-perfluorooctanol was purchased from abcr GmbH (Karlsruhe, Germany). Developer mr-Dev 600 was purchased from micro resist technology GmbH (Berlin, Germany). Sylgard^®^ 184 Elastomer Kit was purchased from Biesterfeld Spezialchemie GmbH (Hamburg, Germany).

NMR spectra were measured on a Bruker Avance III 500. For the photolithographic preparation of microfluidic devices, masters were prepared from SU−8 2015 (micro resist technology GmbH, Berlin, Germany) using a MJB3 mask aligner (Süss MikroTec, Garching, Germany). For the preparation of W/O emulsions, microfluidic devices were connected to high-precision syringe pumps (low-pressure syringe pumps neMESYS 290 N, Cetoni, Germany). Microfluidic experiments were followed on inverted brightfield microscopes (Axio Vert.A1 or Primovert; both Carl Zeiss Microscopy GmbH, Jena, Germany) equipped with Phantom Miro C110 or Miro eX4 high-speed digital camera (both Vision Research Inc., Wayne, NJ, USA). For on-chip UV-polymerization, a UV-source (OmniCure^®^ S1500, Asslar, Germany) was connected to the Primovert brightfield microscope (Carl Zeiss Microscopy GmbH, Munich, Germany) by a homemade optical pathway, as further detailed in Figure S11. Phase-contrast and fluorescence microscopy imaging was performed on a Leica DMi8 (Wetzlar, Germany). Confocal microscopy images were recorded on an Andor Dragonfly spinning confocal microscope equipped with an iXon Ultra 888 camera (Andor Technology Ltd., Belfast, UK) and on a Leica TCS SP8 confocal microscope (Wetzlar, Germany). Mechanical characterization of microgels was performed using real-time deformability cytometry (RT-DC) [27] in an AcCellerator (Zellmechanik Dresden, Dresden, Germany). Briefly, the experimental setup is built around a microfluidic chip with a constriction of 300 µm length and a squared cross-section of 40 × 40 µm. Measurements were performed at a flow rate of 0.60 µL s^−1^ and, as a buffer, we used PBS (without Ca^2+^ and Mg^2+^) with 1% (w/v) methyl cellulose (MC-PBS).

### 2.2. Screening of HA-PDPH Synthesis via EDC- and DMTMM-Activation

With slight modifications, PDPH-modified HA was prepared via EDC-activation according to the synthesis reported by Young et al. [28]. Briefly, to a solution of sodium hyaluronate (20 mg, 0.050 mmol) in 8 mL of 0.1 g mol^−1^ MES buffer (pH 4.75), EDC (38 mg, 0.198 mmol), NHS (1 mg, 0.009 mmol), and PDPH (5.9 mg, 0.0248 mmol) were stepwise added and stirred at room temperature (RT). At selected time points (2, 4, 6, 8, 24, 120 h), the reaction mixtures were extensively dialyzed (3.5 kDa MWCO) against deionized water for two days and subsequently lyophilized to yield HA-PDPH.

For HA-PDPH synthesis via DMTMM activation, DMTMM (54.9 mg, 0.198 mmol) and PDPH (5.9 mg, 0.0248 mmol) were added to a solution of sodium hyaluronate (20 mg, 0.050 mmol) in 0.1 g mol^−1^ MES buffer (pH 5.5). After distinct time points (2, 4, 6, 8, 24, 120 h), consecutive purification and characterization were performed analogous to the synthesis of HA-PDPH via EDC-activation.

^1^H-NMR (500 MHz, D_2_O): δ = 8.5 ppm (s, 1H), 8.0 ppm (s, 2H), 7.4 ppm (s, 1H), 4.8–3.2 (HA-backbone), 3.2 ppm (s, 2H), 2.9 ppm (s, 2H), 2.1 ppm (m, 3H). Degree of substitution (DS) (PDPH via DMTMM) = 4–40%. DS (PDPH via EDC) = 25–27%.

### 2.3. Synthesis and Characterization of HA-Derivates

For all HA-derivate syntheses, sodium hyaluronate (100 mg, 0.248 mmol) was dissolved in 0.1 g mol^−1^ MES-buffer (pH 5.5) and supplemented with DMTMM (206 mg, 0.744 mmol). Upon DMTMM-activation, the coupling reagents were added dropwise in molar ratios, as listed in Table 1. Due to insolubility in aqueous solutions, DBCO-amine was added in pure DMSO giving a final water-DMSO mixture of 4:1 (v/v). After 5 days of reaction, all reaction mixtures were extensively dialyzed (10 kDa MWCO) against aqueous 0.1 M NaCl for 2 days and another 2 days against deionized water. Prior to dialysis, PDPH-incubated HA-derivates were reacted with TCEP-HCl (85 mg, 0.372 mmol) overnight to cleave PDPH-disulfides. Finally, the HA-derivates were isolated by lyophilization, and analyzed by ^1^H-NMR.

- HASH: ^1^H-NMR (500 MHz, D_2_O): δ = 4.7–3.2 ppm (HA-backbone), 2.9 ppm (m, 2H), 2.8 ppm (m, 2H), 2.1 ppm (m, 3H). DS (SH) = 13–65%.
- HAmFU: ^1^H-NMR (500 MHz, D_2_O): δ = 6.3 ppm (m, 1H), 6.1 ppm (m, 1H), 4.7–3.2 ppm (HA-backbone), 2.3 ppm (m, 3 H), 2.1 ppm (m, 3 H). DS (mFU) = 13–61%.
- HADBCO via DBCO-amine: ^1^H-NMR (500 MHz, D_2_O): δ = 7.6 ppm (m, 8H), 5.2 ppm (m, 1H), 4.7–3.2 ppm (HA-backbone/DBCO-amine), 2.6 ppm (m, 1H), 2 ppm (m, 4H). DS (DBCO) = 13–20%.
- HADBCO via DBCO-sulfo-PEG_4_-amine: ^1^H-NMR (500 MHz, D_2_O): δ = 7.6 ppm (m, 8H), 5.2 ppm (m, 1 H), 4.7–3.2 ppm (HA-backbone/PEG-DBCO), 2.6 ppm (m, PEG), 2.0 ppm (m, 4H). DS (DBCO) = 4–35%.

### 2.4. Determination of the Gelation Time Based on Pipetting Studies

Based on a previously reported method [29], the gelation time of HA-hydrogels was estimated by a so-called bulk pipetting test. Briefly, stocks of HA- and PEG-precursor solutions were prepared according to the concentrations as later applied in the fabrication of HA-microgel species (Table 2). To provide crosslinking conditions similar to the microfluidic HA-microgel formation, the HA-based bulk solutions were preincubated with modified-Atto565. For HAmFU- and HADBCO-hydrogel formation, the precursor solutions were mixed 1:1 (v/v) and gently vortexed in an 0.5 mL Eppendorf tube giving a total volume of 40 µL. Over time, the gelation was followed by testing the pipettability using a 10 µL Eppendorf pipette. The gel state was defined as the point, where the uptake of the solution was prohibited by clogging of the pipette tip due to gel formation. In case of HASH-hydrogel gelation studies, the mixed precursor solutions were supplemented with 0.25% (w/v) LAP photoinitiator and UV-irradiated under our brightfield microscope setup (Figure S11) using the same conditions as applied for the preparation of HASH-microgels (n = 4; mean ± SD; measuring intervals: ≈1 s (HASH-hydrogels), 5 s (HADBCO-hydrogels), 60 s (HAmFU-hydrogels)).

### 2.5. Microfluidic Device Fabrication

Poly(dimethylsiloxane) (PDMS)-based microfluidic devices were prepared by combined photo- and soft lithography, as previously described [7]. Briefly, after spin-coating a negative photoresist (SU−8 2015) onto a silicon wafer, the desired CAD-based microstructure was transferred from a photomask into the photoresist using a mask aligner. Non-illuminated parts were removed by washing with mr-Dev 600 developer to yield the structure of a desired microchannel network with defined height and width. Subsequently, a degassed mixture of PDMS oligomer and crosslinker combined at a ratio of 10:1 (w/w) was poured onto the microfluidic master device and polymerized in an oven at 80 °C for 2 h. Upon cutting-out the structures and punching in- and outflow ports into the PDMS replica, the device was bonded to a glass slide by oxygen plasma treatment. Prior to usage, the microchannels were treated with a solution of 0.5% (v/v) trichloro (1*H*,1*H*,2*H*,2*H*-perfluorooctyl) silane in fluorinated oil (HFE−7500) to increase the hydrophobicity of the channel walls.

### 2.6. Microfluidic Preparation of HA-Microgels

For microfluidics-assisted formation of W/O emulsions, a microfluidic flow-focusing device with a channel height and width of 25 µm was employed (Figure 2A). Prior to emulsification, HA-derivates were preincubated with functional Atto565-dyes, as listed in Table 2. Upon labeling of the hydrogel precursor, the HA-derivates and homobifunctional PEG-crosslinkers, both dissolved in 0.1 g mol^−1^ MES buffer (pH 5.5) were co-injected into the microfluidic device and emulsified into droplets by a continuous phase made of a fluorinated oil (HFE−7500), supplemented with 2% (w/w) of a homemade triblock copolymer surfactant [7,30]. The flow rates were set to 500 µL h^−1^ for the continuous phase and 25 µL h^−1^ for both of the dispersed phases. In the cases of HAmFU- and HADBCO-microgel formation, the emulsions were directly collected in an Eppendorf tube, covered with a layer of light mineral oil to prevent the droplets from drying, and allowed to gel overnight. For the UV-initiated synthesis of HASH-microgels, the PEG-containing dispersed phase was additionally supplemented with 0.25% (w/v) LAP photoinitiator. To undergo UV-initiated polymerization, HASH-precursor droplets were injected into a microfluidic on-chip UV-chamber with a uniform height of 50 µm and irradiated by a UV-light source (250–450 nm, 44.6 mW cm^−2^), which was connected to a brightfield microscope via quartz glass optics (Figure S11). For purification, all obtained HA-microgels were transferred into water by performing several washing steps with 20% (v/v) 1*H*,1*H*,2*H*,2*H*-perfluoro−1-octanol (PFO) in HFE−7500, followed by treatment with 0.5% (v/v) Span 80 in hexane, and pure hexane to remove final traces of microgel-entrapped oil residues.

### 2.7. Swelling Studies and Stability Tests of HA-Microgels

The swelling behavior of HA-microgels was calculated as the ratio between the average volume of solvent-swollen HA-microgels and the average volume of corresponding droplet W/O emulsion droplets. Evaluation was conducted based on fluorescence microscopy images of 50 droplets and microgels, respectively, taken into account. For analysis of HA-microgel stability over time, microgels suspended in water or PBS were injected into a sealed microscopy image chamber and stored at 4 °C. At selected time points, fluorescence microscopy images were recorded, and the swelling ratio was calculated.

### 2.8. Thermal Stability Studies

Dissolved in PBS, HAmFU-0.5, HASH-0.5, and HADBCO-0.5 microgel suspensions were transferred into individual 1.5 mL Eppendorf tubes and heated on a thermoshaker to selected temperatures (37, 65, and 90 °C). After distinct time points (5, 15, 30, 60, 90 min), the microgels were directly imaged on a fluorescence microscope, whereby the swelling ratio over time was determined for 50 microgels.

### 2.9. FITC-Dextran Permeability Studies

In PBS, HA-microgels were incubated with FITC-dextran solutions of varying molecular weights (4, 40, 150, 250, 500, 2000 kDa) to give a final concentration of 0.5 mg mL^−1^. After 24 h of continuous shaking, the microgels were imaged via spinning-disc confocal microscopy. The relative microgel permeability was calculated by comparing the fluorescence signal of the particle-surrounding solution to the inside of the microgel network using ImageJ 1.52 t with the Radial Profile Angle plugin (radius = 52 µm corresponding to 80 pixels; integration angle = 45°). For data evaluation, measurements over 15 particles per dextran species were performed, whereby the background signal of the confocal microscope was subtracted.

### 2.10. Real-Time Deformability Cytometry

In a typical experiment, microgels were resuspended in 1% (w/v) MC-PBS buffer at a concentration of approximately 1 million per milliliter. After stabilizing the flow inside the microfluidic chip, several thousand of microgels were characterized per condition. The Young’s modulus has been obtained from an analytical model utilizing cell deformation and size [31]. Statistical significance was obtained from microgel replicates using linear mixed models [32].

### 2.11. Non-Spherical HASH-Microgels

According to the foregoing preparation of HASH-microgels, 3.56% (w/v) HASH was mixed on-chip with a solution of 3.77% (w/v) PEG-norb_2_ containing 0.25% (w/v) LAP. As-formed W/O emulsions were directly injected into an on-chip UV-chamber with a height of 20 µm and UV-polymerized (250–450 nm, 44.6 mW cm^−2^), while being retained in non-spherical shape. Egg-shaped HASH-0.75 microgels were prepared by setting the flow rates to 500 µL h^−1^ for the continuous phase and 30 µL h ^−1^ for both dispersed phases. Disk like HASH-0.75 microgels were prepared by setting the flow rates to 300 µL h^−1^ for the continuous phase and 50 µL h^−1^ for both dispersed phases. The 3D reconstruction of x,y-microgel images and the evaluation of the corresponding microgel aspect ratios were performed using the Leica LAS X 3 D visualization software.

### 2.12. Synthesis of Trifunctional HA(mFU, biotin, DBCO)

In Milli-Q water, biotin-PEG-maleimide (34.4 mg, 0.007 mmol) was added dropwise to a solution of highly methylfuran-substituted HA (50 mg, 0.109 mmol; DS = 63%) and reacted for 24 h. Upon removal of non-coupled reagents by dialysis (10 kDa MWCO) against Milli-Q water for 2 days, the solution was lyophilized to yield HA (mFU, biotin) as a dry powder. To obtain trifunctional HA (mFU, biotin, DBCO), to a solution of biotin-PEG-maleimide-reacted HAmFU, DBCO-PEG_4_-maleimide (4.6 mg, 0.007 mmol) was dropwise added and stirred for another 24 h. Non-coupled reagents were removed by dialysis (10 kDa MWCO) against Milli-Q water for 2 days to yield hetero-trifuntional HA (mFU, biotin, DBCO) after lyophilization.

- HA (mFU, biotin): ^1^H-NMR (500 MHz, D_2_O): δ = 6.3 ppm (m, 1H), 6.1 ppm (m, 1H), 4.7–2.5 ppm (HA-backbone/PEG-biotin), 2.3 ppm (m, 3H), 2.1 ppm (m, 5H), 1.4–1.9 ppm (m, 6H). DS (biotin) ≈ 9.5%.
- HA (mFU, biotin, DBCO): ^1^H-NMR (500 MHz, D_2_O): δ = 7.6 ppm (m, 8H), 6.3 ppm (m, 1H), 6.1 ppm (m, 1H), 5.2 ppm (m, 1H), 4.7–2.5 ppm (HA-backbone/PEG-biotin/PEG-DBCO), 2.3 ppm (m, 3H), 2.1 ppm (m, 5H), 1.4–1.9 ppm (m, 6H). DS (DBCO) ≈ 9%.

### 2.13. MF Preparation and Proof of Availability of Functional Moieties in Trifunctional HA-Microgels

For fabricating hetero-trifunctional HA-microgels, 3.5% (w/v) HA(mFU, biotin, DBCO) was dissolved in PBS and successively incubated with 0.4 µL Atto647 N-azide, 0.3 µL Atto565-azide, and 0.3 µL Atto425-streptavidin (all: c = 1 µg µL^−1^) for 2 h. Applying the same microfluidic device as employed in Section 2.6, preincubated HA(mFU, DBCO, biotin) was mixed on-chip with 2.09% (w/v) PEG-mal_2_ to prepare W/O emulsion by setting the flow rates to 500 µL h^−1^ for the continuous phase and 25 µL h^−1^ for both of the dispersed phases. Consecutive purification of formed microgels was performed following the purification procedure, as described above.

## 3. Results

### 3.1. Synthesis and Characterization of HA-Derivates for Microgel Formation

**Figure 1:**
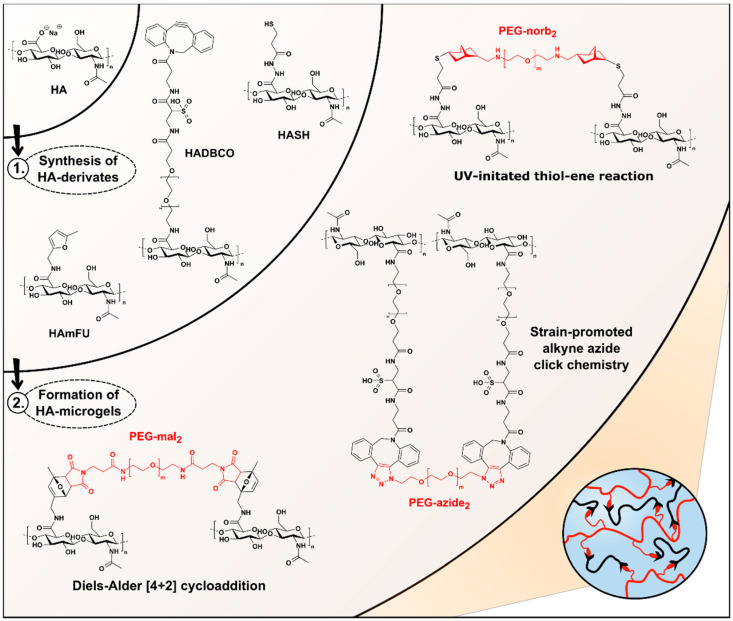
Overview of HA-microgel design based on Diels–Alder [4 + 2] cycloaddition, SPAAC, and UV-initiated thiol–ene reaction. Upon DMTMM-mediated synthesis of HA-derivates, HASH-, HADBCO-, and HAmFU-microgels are microfluidically prepared by crosslinking HASH, HADBCO, and HAmFU with PEG-norb_2_, PEG-azide_2_, and PEG-mal_2_, respectively.

For the design of HA-microgels with well-controlled physicochemical and mechanical properties, we evaluate three different click reactions: UV-initiated thiol–ene reaction [33], Diels–Alder [4 + 2] cycloaddition [34,35], and SPAAC [36,37], which have been widely reported for biomaterial design in bulk (Figure 1). To render the HA-backbone accessible towards covalent crosslinking, most common approaches rely on the attachment of amine- or hydrazide-terminated functionalities to the HA-carboxylates via esterification [21,38]. To cover a broad range of crosslinking densities and thus achieve flexibility in microgel design, distinct control over the degree of substitution (DS) of the carboxylic groups is crucial. On this account, we investigate two common activation reagents regarding their reaction kinetics and efficiencies: EDC and DMTMM. Exemplarily, both DMTMM- and EDC-activated HA-derivatives are incubated with PDPH, which is applied as a coupling reagent in the later-described synthesis of thiol-modified HA (HASH) (Figure S1C and Figure S2) [28]. The degree of PDPH-substitution is monitored via ^1^ H-NMR over time, comparing the integrals of the *N*-acetyl glucosamine protons at 2.1 ppm to the aromatic protons of PDPH at 7.5, 8.0, and 8.5 ppm (Figure S3A,B; Table S1). In agreement with earlier studies [39,40], we find that PDPH-coupling via EDC results in a more rapid amidation, whereas higher DS are yielded over time, when DMTMM is employed (Figure S3C). The lower efficiency of PDPH-substitution via EDC is attributed to the rapid deactivation of the EDC-intermediate in aqueous media [41] and the pH-discrepancy between EDC-activation at acidic conditions (pH 3.5−4.5) [42] and efficient amidation at basic conditions. Moreover, most likely due to electrostatic binding to HA, the remaining urea byproduct [39] is observed, which cannot be removed even by extensive dialysis and may thus interfere with the consecutive conjugation of bioactive substances.

Based on these preliminary results, all consecutive HA-modifications are performed via DMTMM-mediated activation following the synthesis routes depicted in Figure S1. To screen the range of DS, which are accessible, the stoichiometric ratios of the coupling reagents are systematically varied (Table 1). As mentioned before, HASH is prepared by PDPH-coupling via DMTMM, followed by TCEP treatment to give thiolated HA upon disulfide cleavage (Figure S1C). The DS of HASH is determined by comparing the signal of *N*-acetyl glucosamine protons at 2.1 ppm to the peaks arising from the methylene groups at 2.8 ppm and 2.9 ppm (Figure S4). By varying the equivalents of PDPH, a broad range of HA-backbone substitutions between 13% and 65% are obtained. Similar results with DS-values ranging from 13% to 61% are found, when 5-methylfurfurylamine is introduced as a coupling reagent to yield methylfuran-modified HA (HAmFU) (Figure S1A). Here, the methylfuran-substitution is confirmed by the presence of NMR resonances at 6.1 ppm and 6.3 ppm, referring to the aromatic furan protons, which are compared to the protons of *N*-acetyl glucosamine at 2.1 ppm (Figure S5). Initial attempts to prepare dibenzocyclooctyne-modified HA (HADBCO) are performed by reacting HA with DBCO-PEG_4_-amine in DMSO-water mixture. The signals of aromatic cycloalkyne protons at 7.6 ppm are related to HA-ring protons at 4.6 ppm (Figure S6). Employing 0.125 Eq and 0.25 Eq of DBCO-amine results in HADBCO-derivates with DS of 13% and 20%, respectively, which reveal an overall poor solubility in aqueous media. Notably, inserting DBCO-amine concentrations above 0.25 Eq results in prominent precipitation of HA during synthesis. Due to an overall lack of sufficient solubility in aqueous media, HADBCO can only be processed via W/O emulsion droplets into microgels at minimal concentrations. However, especially in the case of microfluidic on-chip mixing of two polymer precursors—to prevent uncontrolled gelation as compared to premixed precursor solutions—concentrated precursor solutions are required to account for the dilution by the other injected precursor stream. To overcome this major hurdle and improve the solubility of the HADBCO precursor, DBCO-sulfo-PEG_4_-amine is selected as an alternative coupling reagent providing enhanced water solubility due to its additional sulfo-modification (Figure S1B). Compared to the results obtained for HASH- and HAmFU-derivate synthesis, the DS of this approach is yet limited, ranging between 4% and 35% (Figure S7). We hypothesize that due to the comparably higher molecular weight of DBCO-sulfo-PEG_4_-amine, steric hindrance may lead to lower reactivity towards the HA-carboxylates.

### 3.2. Droplet Microfluidics-Assisted Fabrication of HA-Microgels

#### 3.2.1. Gelation Properties of HA-Microgels Based on Bulk Pipetting Studies

To guarantee stable microfluidic processing of HA and PEG into droplets for microgel formation via three different crosslinking strategies, the microchannel design needs to account for the gelation rates of the respective type of click reaction regarding mixing and dwell time of the precursor solutions. To assess the gelation kinetics, in bulk, pipettability tests over time are performed by mixing the polymer precursor solutions at the same concentrations as for the MF microgel fabrication (Table 2). Depending on the click chemistry mechanism and PEG-crosslinker concentration, we observe a variation in gelation time ranging from 1 s to 64 min (Table 3). While the HA-hydrogel formation via Diels–Alder [4 + 2] cycloaddition proceeds rather slowly, a significant acceleration of the network formation via SPAAC is observed, whereas, in case of UV-initiated thiol–ene reaction, HA-hydrogels are rapidly crosslinked within less than 1 s upon UV-light trigger (250–450 nm, 44.6 mW cm^−2^). For all types of click reactions, with increasing PEG-crosslinker concentration, accelerated gelation kinetics are observed, which we attribute to the higher amounts of functional moieties available to undergo chemical crosslinking. Notably, increasing the PEG-crosslinker content or altering the type of click chemistry not only influences the kinetics of hydrogel formation, but also affects the physicochemical properties of HA-microgels, as further detailed in Section 3.3.

#### 3.2.2. Microfluidic Formation of Microgel Precursor Droplets

**Figure 2:**
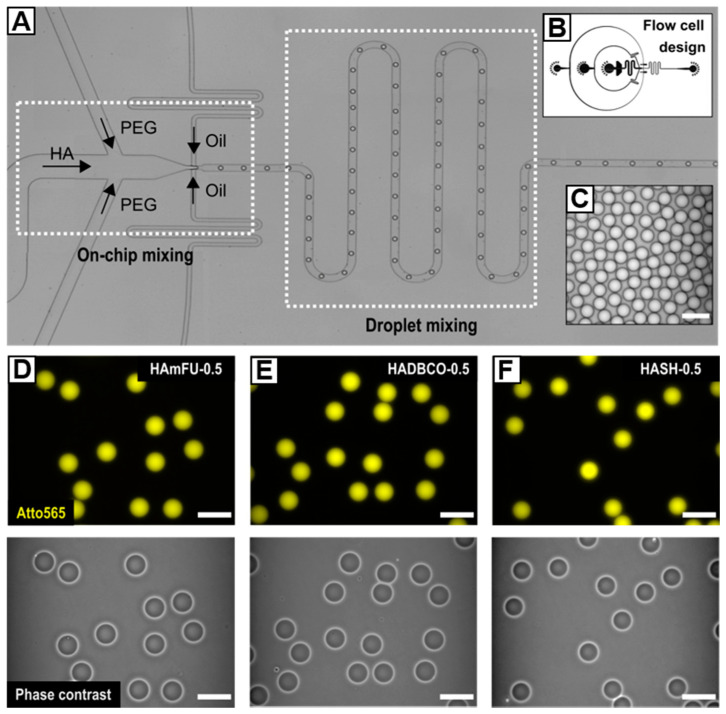
(**A**) Microfluidic flow-focusing device for on-chip mixing of HA-derivates with homobifunctional PEG-crosslinkers. The cross-junction is connected to a serpentine meander structure that induces fast and efficient droplet mixing. (**B**) Corresponding CAD-based schematic of the flow cell. (**C**) Exemplarily W/O emulsion showcasing the preparation of uniform droplets. (**D**–**F**) HA-microgels in PBS prepared from (**D**) HAmFU, (**E**) HADBCO, and (**F**) HASH via crosslinking with 0.5 Eq of homobifunctional PEG-crosslinkers. Upper row: Fluorescence microscope images of HA-microgels labeled with Atto565 (yellow). Bottom row: Corresponding phase-contrast images. All scale bars denote 50 µm.

Considering the results of preceding bulk gelation studies, we introduce a microfluidic flow cell design, which enables separate injection of two microgel precursor components to spatiotemporally confine the click reaction exclusively to the droplet volume (Figure 2). With that, the design accounts for the nature of fast gelling click reactions, where injecting microgel precursors as a premixed dispersed phase would result in bulk gelation inside the syringe or the flow cell prior to droplet formation. Upon droplet pinch-off, a subsequent serpentine meander structure promotes fast and efficient mixing of HA and PEG through chaotic advection yielding a homogenous distribution of microgel precursors throughout the droplet volume [43,44]. To prevent the collected W/O emulsions from coalescence, the fluorinated oil phase (HFE−7500) is supplemented with 2% (w/w) of a homemade triblock copolymer surfactant consisting of Krytox^®^–Jeffamine^®^–Krytox^®^ [7,30].

The range of accessible droplet diameters at a fixed microchannel height and junction width of 25 µm is determined by screening the flow characteristics of the microfluidic device. As depicted in Figure S8, depending on the flow rate ratio *q*, which we define as the ratio between the flow rates of the continuous phase (*Q*_c_) and the dispersed phases (*Q*_d_), droplets with sizes ranging from 47.1 ± 0.9 µm to 24.5 ± 0.7 µm can be processed. As expected, the droplet diameter increases with decreasing *q*, eventually shifting the flow pattern from a dripping to a jetting regime [45]. While stable co-flow of continuous and dispersed phases appears when *q* is set below 1.5, we set *q* to 10 to ensure stable droplet formation and hence long-term and quantitative microgel fabrication.

HA-microgels are obtained by crosslinking HAmFU with PEG-dimaleimide (PEG-mal_2_; 5000 g mol^−1^) via Diels–Alder [4 + 2] cycloaddition, HADBCO with PEG-diazide (PEG-azide_2_; 5000 g mol^−1^) via SPAAC, and HASH with PEG-dinorbornene (PEG-norb_2_; 6000 g mol^−1^) via UV-initiated thiol–ene reaction (Figure 1). All W/O emulsions are formed by setting the flow rates to 500 µL h^−1^ for the continuous phase and 25 µL h^−1^ for each of the two dispersed phases. At these flow rates, uniform droplets—25 µm in diameter on average—are obtained (Table 4), whereby minor differences in diameter result from slight variations in fluid viscosity among the injected precursor solutions.Since microgels generally reveal low-scattering contrast in brightfield microscopy, prior to injection, HA-derivates are fluorescently labeled with Atto565-dyes, which are attached to the same functional moieties as applied for microgel network formation. Notably, due to an excess of respective functionalities attached to HA, low quantities of Atto565 do not limit the successive crosslinking with PEG.

To investigate the dependence of the HA-microgel’s physicochemical and mechanical properties on the crosslinking density and type of crosslinking reaction (see Section 3.3), PEG concentrations are varied from 0.5 Eq to 0.75 Eq (Table 2). Notably, to provide comparable experimental conditions regarding the number of available functional moieties for crosslinking, HA-derivates of similar DS are applied with 27% for HAmFU, 23% for HADBCO, and 24% for HASH.

#### 3.2.3. Preparation of HAmFU-Microgels via Diels–Alder [4 + 2] Cycloaddition

The Diels–Alder [4 + 2] cycloaddition proceeds highly specific through the reaction of a diene and a dienophile without the need for an initiator or catalyst [46]. As reported by the Shoichet group [34,35], by chemically modifying HA with either furan- or methylfuran-moieties, bulk hydrogels can be fabricated by crosslinking with PEG-mal_2_ to engineer biomaterials for 3D cell culturing. Furthermore, it has been shown that introducing a methyl-group to furan results in accelerated gelation kinetics, which overall renders HAmFU more suitable for fast and tailored microgel design than furan-modified HA. Following the above-described microfluidic approach to form HA-microgels, W/O emulsions composed of HAmFU and PEG-mal_2_ are collected in an Eppendorf tube, allowed to gel overnight at RT, and transferred into water by breaking the emulsion with 20% (v/v) PFO in HFE−7500. Notably, after 12 h of gelation, we observe a dry-out of the W/O emulsion at the emulsion-air interface shifting the droplet size distribution towards smaller diameters (Figure S9A). As a protective layer against droplet evaporation, we thus overlay collected W/O emulsions with light mineral oil, by which means droplet monodispersity is preserved during gelation (Figure S9B).

#### 3.2.4. Preparation of HADBCO-Microgels via SPAAC

Copper(I)-catalyzed azide-alkyne click (CuAAC) reactions have been applied for preparing HA-based hydrogel networks for diverse bioapplications [47,48,49]. However, upon click reaction, cytotoxic copper traces may remain inside the polymer network, harming encapsulated bioactive compounds, such as DNA, proteins, or cells [4,50]. Addressing these drawbacks, due to its high reactivity and efficiency, we focus on the SPAAC reaction, enabling hydrogel formation at ambient temperatures without further catalysis. As shown by other research groups [36,37], bulk hydrogels can be prepared by crosslinking cyclooctyne-modified HA-derivates with azide-modified PEG- or HA-precursors providing high cell compatibility. Therefore, we introduce SPAAC for microgel formation by crosslinking HADBCO with PEG-azide_2_ following the same microfluidic approach as described for the preparation of HAmFU-microgels (see Section 3.2.2).

#### 3.2.5. Preparation of HASH-Microgels via UV-Initiated Thiol–Ene Reaction

**Figure 3:**
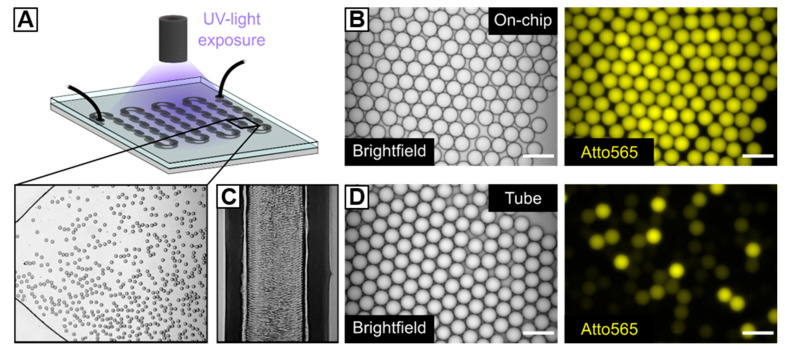
Microfluidic fabrication of HASH-microgels. (**A**) Schematic depiction of a microfluidic chamber for UV-initiated in situ crosslinking of HASH-based W/O emulsion droplets under continuous flow and corresponding brightfield image. (**B**,**D**) Brightfield images and fluorescence microscopy images (Atto565) of W/O emulsion droplets after UV-irradiation (B) within the microfluidic on-chip UV-chamber with a height of 50 µm and (D) within the microfluidic outflow tubing of the flow-focusing device. (**C**) Densely packed W/O emulsion inside the outflow tubing of the flow-focusing device. The scale bars denote 50 µm. The experimental microscope setup for the on-chip UV-polymerization of HASH-based W/O emulsions is depicted in Figure S1.

Thiol–X reaction manifests itself through high and rapid reactivity under mild and physiological reaction conditions via various pathways—e.g., thiol-halide, thiol-ene, or thiol-isocyanate click reaction [51,52]. Among the many approaches to realize thiol–ene-mediated material designs, photoinitiators, e.g., Irgacure^®^ 2959 or LAP have been used as a radical source, since they ensure spatiotemporal control over hydrogel formation by a UV-light trigger. For instance, Gramlich et al. [33] have highlighted the high specificity of UV-initiated thiol-norbornene click reaction by preparing cytocompatible, photopatterned bulk hydrogels based on norbornene-modified HA and di-thiols with tunable mechanical properties. We transfer thiol–ene reaction into a HASH-microgel system by UV-crosslinking HASH with PEG-norb_2_ in the presence 0.25% (w/w) LAP as a photoinitiator. When the outflow tubing of the flow-focusing device is exposed to UV-light–to induce microgel formation–pronounced bleaching of emulsion droplets and Atto565-dye, respectively, is observed (Figure 3D). This may be due to inconsistent irradiation within the densely packed tubing (Figure 3C), thus leading to heterogeneity in microgel crosslinking density. We hypothesize that droplets arrange in layers over each other resulting in differences in UV-exposure energy and time. On this account, directly after their formation, W/O emulsions are transferred into an on-chip UV-chamber with a height of 50 µm, whose structural design allows for preserving formed droplets as a monolayer while being exposed to UV-light (250–450 nm, 44.6 mW cm^−2^) (Figure 3A). By this means, droplet emulsions of narrow size distribution are obtained, although slight bleaching of HASH-conjugated Atto565-dye still appears (Figure 3B). Notably, when LAP is replaced against 0.25% (w/v) Irgacure^®^2959, no microgel formation is observed. This effect is attributed to the lower solubility of Irgacure^®^2959 in water as compared to LAP, suggesting the diffusion through the dynamic surfactant-stabilized W/O interface into the surrounding oil phase [53,54].

### 3.3. Characterization of HA-Microgel Porosities

#### 3.3.1. HA-Microgel Swelling Properties and Stability in Aqueous Media

**Figure 4:**
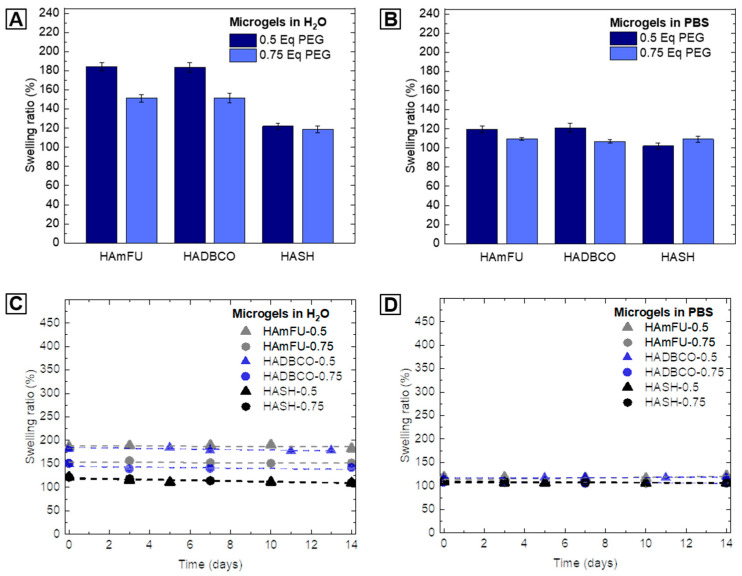
Swelling behavior and stability of HA-microgels in aqueous media. (**A**,**B**) Swelling ratio of HA-microgels in (A) water and (B) PBS related to the amount of PEG-crosslinker used for microgel formation (n = 100; mean ± SD). (**C**,**D**) Analysis of microgel stability in (C) water and (D) PBS over time (n = 50; mean ± SD). The swelling ratio is defined as the ratio between the average diameter of W/O emulsion droplets and the average diameter of obtained HA-microgels after purification.

We determine the swelling behavior of HA-microgel species in water and PBS, whereby the swelling ratio is defined as the ratio of the average diameter of as-prepared droplets and the corresponding HA-microgel average diameter after swelling to equilibrium in the respective medium (Table 4; Figure 4A,B). By varying the amount of PEG-crosslinker from 0.5 Eq to 0.75 Eq, thus increasing the crosslinking density, a decrease in swelling is observed. Since HA-hydrogels highly respond to changes in pH, ionic strength, or temperature of their surrounding environment, we attribute the minor swelling in PBS to the presence of salt ions, which electrostatically affect the HA-network structure [55,56]. Importantly, the effect of PEG-crosslinker concentration on swelling behavior is found to vary between the three types of click chemistry-mediated HA-microgels studied in here. Microgels based on HAmFU and HADBCO reveal an overall higher degree of swelling than those prepared from HASH. Moreover, in contrast to HAmFU- and HADBCO-microgels showing similar response to PEG-crosslinker variations, HASH-microgels exhibit no significant difference in swelling with regards to the crosslinker concentration. From these results, we interpret the microgel network structure not only to be affected by the polymer content itself but also to be related to the reactivity of involved click chemistry reagents, and hence their efficiencies in crosslinking. 

To further investigate the long-term stability of HA-microgels in aqueous solutions, microgel suspensions are stored at 4 °C in sealed chambers and imaged over time by fluorescence microscopy. The microgel degradation can be deduced from an increase in microgel swelling and hence, the degradation of the covalently crosslinked microgel network. Yet, at slight acidic (H_2_O; pH ≈ 5) as well as physiological (PBS; pH = 7.4) pH, no further swelling is exhibited, and all microgel populations are sufficiently stable over at least 14 days (Figure 4C,D).

#### 3.3.2. Thermal Stability Studies

**Figure 5:**
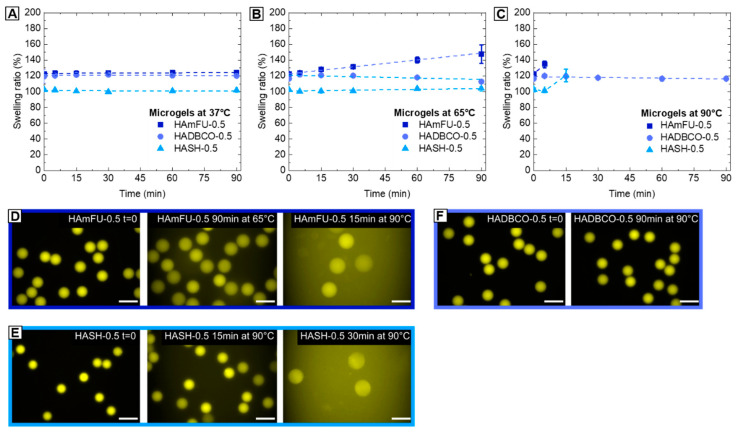
Thermostability studies of HA-microgels crosslinked with 0.5 Eq homobifunctional PEGs. (**A**–**C**) Microgels are heated to (A) 37 °C, (B) 65 °C, (C) 90 °C, respectively, and imaged via fluorescence microscopy over time to determine the swelling ratio and tendency of degradation (n = 50; mean ± SD). (**D**–**F**) Exemplary fluorescence microscope images of (D) HAmFU−0.5, (E) HASH−0.5, and (F) HADBCO−0.5 microgels comparing the initial state (t = 0) to microgels at selected time points and temperatures, respectively. The scale bars denote 50 µm.

When microgel platforms are applied as spatially confined microenvironments, e.g., to perform enzymatic cascade reactions or microgel-based polymerase chain reactions (PCRs), their resistance against thermal degradation is an essential prerequisite to ensure that the polymer network is maintained, and does not change and potentially alter diffusivity inside its microgel volume over time. To compare HA-microgels based on altering crosslinking strategies with respect to their stability at elevated temperatures, HAmFU−0.5, HASH−0.5, and HADBCO−0.5 microgel suspensions are heated to selected temperatures (37, 65, 90 °C) and imaged over time by fluorescence microscopy. As shown in Figure 5, all types of click chemistry-mediated HA-microgels retain their initial dimensions when exposed to 37 °C. While HASH−0.5 and HADBCO−0.5 remain stable at 65 °C, progressing degradation of HAmFU–0.5 is observed. We relate this effect to the retro Diels–Alder reaction becoming more dominant at temperatures above approximately 60 °C [57]. At 90 °C, surprisingly, HADBCO−0.5 remains stable for at least 90 min, whereas HAmFU−0.5 and HASH−0.5 completely degrade and disappear over 5−15 min and 15−30 min, respectively, at this temperature.

#### 3.3.3. FITC-Dextran Permeability Studies

**Figure 6:**
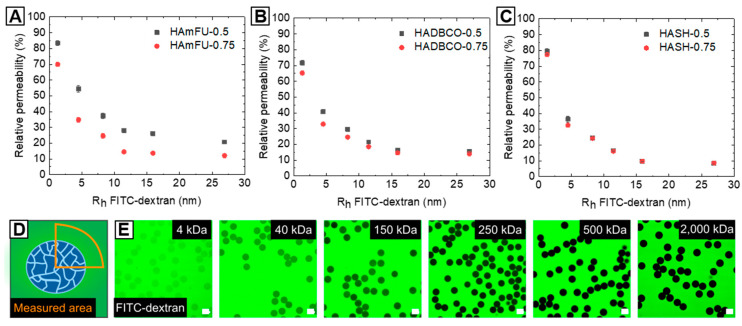
Analysis of HA-microgel permeabilities based on the diffusion of FITC-dextrans of different molecular weights into the microgel networks. (**A**–**C**) Plots of relative permeability versus hydrodynamic radii of FITC-dextrans. Relative permeability of (**A**) HAmFU-microgels, (**B**) HADBCO-microgels, and (**C**) HASH-microgels determined by comparing fluorescence intensity between the microgel volume and the surrounding media, as schematically illustrated in (**D**) (n = 15; mean ± SD). The molecular weights of FITC-dextran are correlated to their hydrodynamic radii in PBS [65,66], as detailed in Table S2. (**E**) Exemplary confocal microscope images of FITC-dextran-incubated HAmFU−0.5 microgels. The scale bars denote 50 µm.

The permeability of solvent-swollen microgels dictates the diffusion of (macro-)molecules—e.g., therapeutic drugs or enzymes into the polymer network—which can then act as carrier systems in drug delivery or for biochemical reactions, such as enzymatic cascade reactions [58,59]. In this context, the quantity of mass transport into microgel networks, which is related to the mesh size between crosslinked polymer chains and their spatial distribution throughout the hydrogel network is of particular interest [60]. However, contrary to an idealized gel topology with uniform pore sizes and absence of any defects, common polymer network structures are composed of multiple structural inhomogeneities arising, e.g., from dangling ends, overlapping chains, loops, or the polymerization mechanism itself [61,62]. For instance, it has been shown that the polymer network formation from prepolymers allows for enhanced control over network homogeneity, as compared to free-radical monomer and crosslinker copolymerization [63]. Yet, compared to well-defined end-to-end-crosslinked networks based on synthetic macromolecules—such as PEG [64]—we assume that side-to-end crosslinking of HA-derivates with homobifunctional PEGs results in more complex and heterogeneous topologies. Contrary to PEG, HA as a natural occurring polymer exhibits broad molecular weights (here: 41–65 kDa), whereby functional moieties that undergo covalent crosslinking are randomly distributed throughout the HA-backbone. To investigate the permeability trends of HA-microgel species depending on the PEG-crosslinker concentration and type of click reaction, the diffusional behavior of a model macromolecule—fluorescein isothiocyanate-labeled dextran (FITC-dextran)—into our HA-microgels is screened by spinning disc confocal microscopy (Figure 6E). In PBS, HA-microgels are incubated with FITC-dextrans for 24 h of various molecular weights (4, 40, 150, 250, 500, 2000 kDa), which are correlated to their hydrodynamic radii [65,66], as summarized in Table S2. 

The microgel permeability is determined by relating the fluorescence intensities inside the particles to the surrounding environment, as schematically illustrated in Figure 6D. As expected, all HA-microgels generally exhibit high accessibility towards smaller molecules, whereas the diffusivity into the microgel network decreases with increasing molecular weight, and thus hydrodynamic radii of FITC-dextrans (Figure 6A–C). Surprisingly, variations in PEG-crosslinker concentrations affect the HA-microgel permeability to a different extent with regards to the type of click reaction. While, in the case of HAmFU-microgels, prominent variations in permeability are found over the range of screened FITC-dextrans (4–2000 kDa), HADBCO-microgels only reveal slight differences in diffusivity of low molecular weight FITC-dextrans (<250 kDa). Compared to that, HASH-microgels show a comparatively poor permeability independent of the FITC-dextran size. Among the numerous complex processes that are subscribed to affect the polymer network topology [60,62] and thus, the microgel permeability, from these results, discrepancies between the types of click reaction are suggested to be related to their gelation kinetics and efficiencies, respectively. Considering previous bulk gelation studies, the network formation is accelerated from HAmFU < HADBCO < HASH, by which means we assume that the polymer precursors can arrange spatially more homogeneous within slower gelling systems giving more uniform networks and hence higher permeabilities. Also, variations in crosslinking efficiency might affect the density of the HA-microgel networks. However, to get a more in-depth insight into the complex physics of HA-microgel formation, extensive analytical studies are required, which are not in the focus of this material guide.

#### 3.3.4. Elasticity

**Figure 7:**
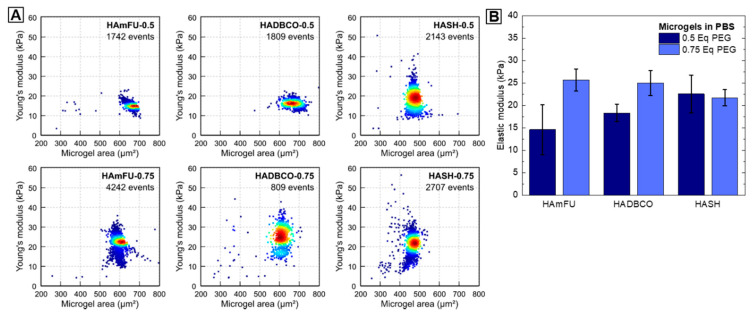
Mechanical characterization of microgels. (**A**) Exemplary Young’s modulus versus microgel area scatter plots for HAmFU-, HADBCO-, and HASH-microgels at PEG-crosslinker concentrations of 0.5 Eq and 0.75 Eq, respectively. Measurements are performed using RT-DC inside a 300 µm long channel of 40 × 40 µm cross-section and at a flow rate of 0.60 µL s^−1^. (**B**) Corresponding statistical analysis of three microgel replicates per microgel species (mean ± SEM).

For engineering microgel-based ECMs for cell culture applications, the elasticity is of key interest to adapt mechanobiological material properties to tissue elasticities found in vivo [67]. In MC-PBS, we determine the elasticity of HA-microgels by performing real-time deformability cytometry (RT-DC) [27]. While RT-DC has been mainly used to study cells [68,69,70], for the first time, we evaluate HA-microgel mechanical properties at high throughput with single-particle resolution (Figure 7A). With regards to variations of PEG-crosslinker equivalents, the Young’s moduli of HA-microgel species reveal similar trends as compared to foregoing swelling and permeability studies (Figure 7B). In case of HAmFU- and HADBCO-microgels, an increase of Youngs’ moduli from 14.6 ± 5.6 kPa to 25.7 ± 2.4 kPa and 18.3 ± 1.9 kPa to 25.0 ± 2.8 kPa, respectively, is observed when the PEG-crosslinker content is increased from 0.5 Eq to 0.75 Eq. This effect can be subscribed to the higher amounts of potential crosslinking sites available, forming denser polymer networks of higher stiffness. In contrast, both HASH-microgel species reveal similar Youngs’ moduli of 22.6 ± 4.2 kPa and 21.7 ± 1.8 kPa. We hypothesize that due to the fast gelation kinetics of thiol–ene-mediated HASH-microgel formation (≤1 s), an increase of PEG-crosslinker content may result in a pronounced formation of elastically inactive network connectivity defects—e.g., dangling ends and loops—rather than in a denser precursor crosslinking.

### 3.4. Preparation of Non-Spherical HASH-Microgels

**Figure 8:**
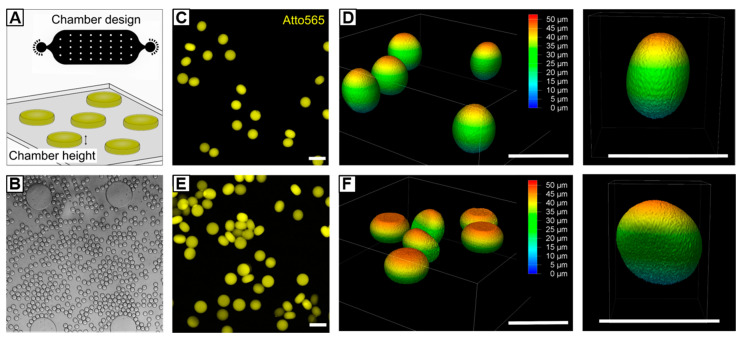
Microfluidic fabrication of non-spherical HASH−0.75 microgels. (**A**) By transferring HASH−0.75 W/O emulsion into a chamber with a height of 20 µm, droplets are compressed into non-spherical shape while being exposed to UV light. Depending on selected flow rates and droplet dimensions, respectively, microgels of different architectures can be generated. (**B**) Exemplary brightfield microscopy image of squeezed droplets flowing through the on-chip UV-chamber. (**C**–**F**) Confocal microscopy images and corresponding z-stacks of (**C**) and (**D**) egg-shaped microgels with an aspect ratio of 1.2 and (**E**,**F**) disk-like microgels with an aspect ratio of 1.5. All scale bars denote 50 µm.

Microgels of tailored asymmetric architecture are of great interest, e.g., to investigate cellular reaction-diffusion pathways in vitro, or to design artificial scaffolds for tissue regeneration [71,72,73]. As shown by others [71,74,75], in situ gelling of droplets with dimensions beyond the height and/or width of a microfluidic channel can be utilized to form non-spherical microgels, such as disks or rods. The goal in these experiments is to manipulate the spherical form of droplets, which they adopt in their thermodynamic equilibrium, by confinement into a desired structure by the surrounding microchannel walls. While most asymmetric microgel systems rely on synthetic monomers or macromers, we show that the fast gelation kinetics of UV-initiated HASH-microgel formation offers new possibilities to prepare HA-microgels of a variety of shapes. For that, HASH−0.75 W/O emulsions are prepared using the same microfluidic device as described in Section 3.2.2 and subsequently transferred into an on-chip UV-chamber with a height of approximately 20 µm (Figure 8A,B). By varying the flow rates of continuous and dispersed phases, the droplet volumes are manipulated towards diameters beyond the chamber height, whereby the droplets are squeezed into non-spherical shapes. After on-chip crosslinking, HASH-microgels are purified and swollen in PBS. To analyze the HASH-microgel shapes depending on the droplet deformation, 3D-imaging via confocal microscopy is performed. As depicted in Figure 8C,D, by setting *q* to 8.3 (*Q_c_* = 500 µL h^−1^; *Q_d_* = 2 × 30 µL h^−1^), droplets are slightly deformed during chamber-confined UV-polymerization giving egg-shaped microgels with an aspect ratio of 1.2. Decreasing *q* to 3 (*Q_c_* = 300 µL h^−1^; *Q_d_* = 2 × 50 µL h^−1^) results in higher compression of the droplet volume with respect to the chamber geometry giving disk-shaped microgels with an aspect ratio of 1.5 (Figure 8E,F). In both microgel systems, minor variations in aspect ratio can be attributed to slight deviations in chamber height and droplet shearing by the chamber walls during flow-through. However, as presented, by controlling the droplet volume and dimensions of the chamber device, microgels of varying architectures beyond spherical shapes can be realized.

### 3.5. Trifunctional HA-Microgels

**Figure 9:**
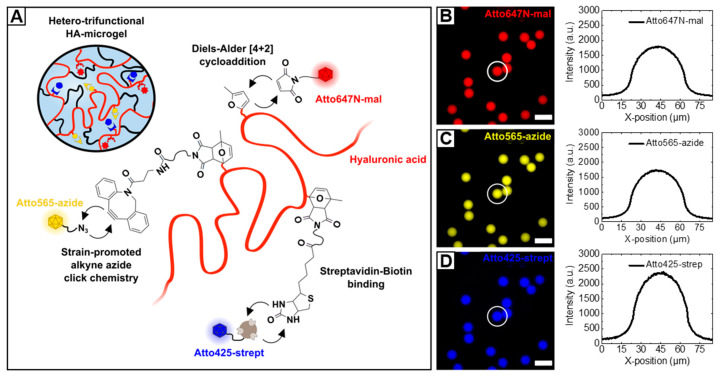
(**A**) Schematic depiction of hetero-trifunctional HA-microgels. By covalently crosslinking methylfuran-moieties with PEG-mal_2_, HA-microgels are formed, whereby remaining methylfurane groups are employed for fluorescent labeling. To proof the availability of biotin- and DBCO-functionalities towards parallel, selective coupling reactions, the HA-microgels are preincubated with Atto425-streptavidin and Atto565-azide dyes via streptavidin-biotin binding and SPAAC, respectively. (**B**–**D**) Fluorescence microscopy images of hetero-trifunctional HA-microgels (27.6 ± 0.9 µm; *n* = 100; mean ± SD) incubated with (**B**) Atto647 *N*-maleimide, (**C**) Atto565-azide, and (**D**) Atto425-streptavidin. Corresponding line scans of individual microgels indicating the homogeneous distribution of the dyes throughout the microgel volume. The scale bars denote 50 µm.

The need for more complex biomaterials that specifically address mechanical, physicochemical, or functional properties has pushed the ongoing development of multifunctional or smart materials beyond a simply crosslinked hydrogel networks [76]. In this perspective, we highlight the versatility of the combination of HA-based material design, microfluidic processing and click reactions by introducing novel hetero-trifunctional HA-microgels. To avoid potential off-target cross-reactions between multiple binding sites—attached to the HA-backbone—functional moieties are incorporated that allow for selective coupling via Diels–Alder [4 + 2] cycloaddition, streptavidin–biotin interaction, and SPAAC (Figure 9A). As base material for microfluidic microgel fabrication, highly methylfuran-substituted HAmFU (DS = 63%) is partially modified with maleimide-PEG-biotin and maleimide-PEG-DBCO in a two-step synthesis. Here, the intermediate PEG-spacers are expected to enhance the accessibility of the functional groups towards subsequent conjugation reactions. After each incubation step, non-coupled PEG-reagents are removed by extensive dialysis, and the products are analyzed by ^1^ H-NMR (Figure S10). The appearance of characteristic protons of biotin-derivate at 1.4–1.8 ppm and 2.8–3.1 ppm, and the presence of aromatic DBCO-protons at 7.6 ppm verify the success of biotin-/DBCO-coupling to the HA-backbone. It is worth noting that, due to multiple overlapping of proton signals arising from both coupling species, the degrees of biotin- and DBCO-modification, respectively, are assessed after each reaction step by comparing the signals of unreacted methylfuran-protons to the initial signal (6.1 ppm and 6.3 ppm) giving a DS of approximately 9.5% for biotin-modification and a DS of approximately 9% for DBCO-modification. To prove the availability of HA-microgel incorporated DBCO- and biotin-groups for conjugation, 3.5% (w/w) hetero-trifunctional HA-precursor is preincubated with Atto647 N-maleimide, Atto565-azide, and Atto425-streptavidin and subsequently on-chip mixed with 2.09% (w/w) PEG-mal_2_ to yield microgels with a diameter of 27.6 ± 0.9 µm in PBS. As verified by fluorescence microscopy imaging, for all three coupling strategies, homogeneous dye distribution throughout the HA-microgel network is observed (Figure 9B–D). In the case of streptavidin-biotin coupling, the slight appearance of Atto425-agglomerates is most likely attributed to electrostatic effects that arise from HA-streptavidin interaction.

## 4. Conclusions

A holistic material guide has been implemented that highlights the synergy of click chemistry strategies and microfluidic microgel fabrication for applications in biosciences. To make HA available for covalent crosslinking, different chemical modifications of HA-carboxylates were screened to enable the microfluidic formation of HA-microgels by Diels–Alder [4 + 2] cycloaddition, SPAAC, and UV-initiated thiol–ene reaction via crosslinking with homobifunctional PEGs. HA-microgels of uniform size were obtained by introducing a tailored microfluidic flow-focusing device that allows for processing even rapidly gelling precursor systems, polymerizing within seconds. By transferring the as-prepared W/O emulsions into an on-chip UV-chamber, thiol–ene-mediated microgel formation was conducted in situ. Here, to provide consistent UV-crosslinking conditions throughout the flow cell, the polymer precursor droplets were passed through the microfluidic chamber in a defined monolayer. In an analytical study, we showed that the HA-microgel’s physicochemical and mechanical characteristics—e.g., swelling behavior, (thermo)stability, permeability, and elasticity were not solely affected by the content of PEG-crosslinker, but also depend on the type of click chemistry mechanism. The modularity of the presented HA-microgel approach serves as a basis to adapt material properties beyond the range of compositions investigated herein, by easily varying the concentration of modified HA- and/or PEG-precursors on-chip or off-chip. To further highlight the versatility of HA-microgel design by droplet microfluidics, the preparation of HASH-microgels with egg- and disk-like shapes was demonstrated by varying microfluidic flow parameters and design features of the flow cell was demonstrated. Moreover, to additionally highlight the potential multi-functionality of our microgels, for the first time, a selective hetero-trifunctional HA-microgel system was developed. As a proof of concept, the availability of all three functional moieties inside the microgels was confirmed by coupling fluorescent streptavidin via biotin-streptavidin interaction, a fluorescent azide via SPAAC, and a fluorescent maleimide via Diels–Alder [4 + 2] cycloaddition to the microgel network. With this bottom-up material guide, we provide detailed insights into the realization of microgels as experimental platforms with tailored material properties, which will simplify their utilization as cell-like biosynthesis environments as well as building blocks in multiphasic gel-in-gel systems, e.g., for advanced cell culturing [13,77].

## Figures and Tables

**Table 1:** Effect of coupling-reagent equivalents on the synthesis of HA-derivates via DMTMM-activation and corresponding DS.

HA-Derivate	Coupling Reagent ^a^	DS
HASH	PDPH (7.1 mg, 0.031 mmol); 0.125 Eq	13%
	PDPH (14.2 mg, 0.062 mmol); 0.25 Eq	24%
	PDPH (28.4 mg, 0.124 mmol); 0.5 Eq	42%
	PDPH (56.9 mg, 0.248 mmol); 1 Eq	65%
HAmFU	5-methylfurfurylamine (3.4 mg, 0.031 mmol); 0.125 Eq	13%
	5-methylfurfurylamine (6.9 mg, 0.062 mmol); 0.25 Eq	27%
	5-methylfurfurylamine (13.8 mg, 0.124 mmol); 0.5 Eq	50%
	5-methylfurfurylamine (27.6 mg, 0.248 mmol); 1 Eq	61%
HADBCO	DBCO-amine (8.6 mg, 0.031 mmol); 0.125 Eq	13%
	DBCO-amine (17.1 mg, 0.062 mmol); 0.25 Eq	20%
	DBCO-sulfo-PEG_4_-amine (16.2 mg, 0.031 mmol); 0.125 Eq	4%
	DBCO-sulfo-PEG_4_-amine (32.5 mg, 0.062 mmol); 0.25 Eq	14%
	DBCO-sulfo-PEG_4_-amine (64.9 mg, 0.124 mmol); 0.5 Eq	23%
	DBCO-sulfo-PEG_4_-amine (129.8 mg, 0.248 mmol); 1 Eq	35%

^a^ Equivalents of coupling reagent are related to the number of moles of HA.

**Table 2:** Summary of employed precursor solutions for HA-microgel fabrication ^a^.

Sample Label	HA-Derivate ^b^	PEG-Crosslinker ^b^	Fluorescent Labeling
HAmFU−0.5	3.50% (w/v) HAmFU ^c^	2.09% (w/v) PEG-mal_2_^d^	0.4 µL Atto565-maleimide ^f^
HAmFU−0.75	3.50% (w/v) HAmFU ^c^	3.14% (w/v) PEG-mal_2_^e^	0.4 µL Atto565-maleimide ^f^
HASH−0.5	3.56% (w/v) HASH ^c^	2.51% (w/v) PEG-norb_2_^d^	0.4 µL Atto565-maleimide ^f^
HASH−0.75	3.56% (w/v) HASH ^c^	3.77% (w/v) PEG-norb_2_^e^	0.4 µL Atto565-maleimide ^f^
HADBCO−0.5	4.66% (w/v) HADBCO ^c^	2.09% (w/v) PEG-azide_2_^d^	0.4 µL Atto565-azide ^f^
HADBCO−0.75	4.66% (w/v) HADBCO ^c^	3.14% (w/v) PEG-azide_2_^e^	0.4 µL Atto565-azide ^f^

^a^ In a typical microfluidic experiment, a total volume of 200 µL of dispersed phase was prepared. ^b^ The concentrations of precursor components refer to the final droplet composition after on-chip mixing. ^c^ n (HA_functional group_ = 3.35 × 10^−3^ mmol). ^d^ n (PEG_functional group_ = 1.68 × 10^−3^ mmol). ^e^ n (PEG_functional group_ = 2.51 × 10^−3^ mmol). ^f^ c = 1 mg mL^−1^.

**Table 3:** Summary of pipettability-based gelation studies.

Sample Label	Gelation Time ^a^
HAmFU−0.5	64 ± 1 min
HAmFU−0.75	58 ± 2 min
HASH−0.5	≤1 s
HASH−0.75	≤1 s
HADBCO−0.5	58 ± 3 s
HADBCO−0.75	26 ± 2 s

^a^ n = 4; mean ± SD; measuring intervals: 5 s (HADBCO-hydrogels), 60 s (HAmFU-hydrogels).

**Table 4:** Emulsion droplet and corresponding microgel diameters depending on variations of crosslinker concentrations and the choice of click reaction.

Sample Label	Eq of Crosslinker	D_droplet_^a^	D_microgel_ (H_2_O) ^a^	Swelling (H_2_O) ^b^	D_microgel_ (PBS) ^a^	Swelling (PBS) ^b^
HAmFU−0.5	0.5 Eq PEG-mal_2_	24.4 ± 0.3 µm	45.0 ± 1.0 µm	184.3 ± 4.1	29.2 ± 0.9 µm	119.6 ± 3.7
HAmFU−0.75	0.75 Eq PEG-mal_2_	26.3 ± 0.3 µm	39.7 ± 1.0 µm	151.2 ± 3.8	28.8 ± 0.4 µm	109.5 ± 1.6
HADBCO−0.5	0.5 Eq PEG-azide_2_	24.6 ± 0.4 µm	45.1 ± 1.2 µm	183.4 ± 4.9	29.8 ± 1.1 µm	121.1 ± 4.6
HADBCO−0.75	0.75 Eq PEG-azide_2_	26.2 ± 0.7 µm	39.7 ± 1.3 µm	151.4 ± 4.9	28.0 ± 0.5 µm	106.8 ± 1.9
HASH−0.5	0.5 Eq PEG-norb_2_	23.7 ± 0.5 µm	28.9 ± 0.7 µm	122.1 ± 3.1	24.2 ± 0.7 µm	102.0 ± 3.2
HASH−0.75	0.75 Eq PEG-norb_2_	24.0 ± 0.7 µm	28.5 ± 0.8 µm	118.6 ± 3.4	26.1 ± 0.9 µm	109.1 ± 3.1

^a^ n = 100; mean ± SD; ^b^ D_microgel_/D_droplet_.

## Supplementary Materials

The following are available online at https://www.mdpi.com/2073-4360/12/8/1760/s1.
