# Supplementary material for: Microfluidic Fabrication of Click Chemistry-Mediated Hyaluronic Acid Microgels: A Bottom-Up Material Guide to Tailor a Microgel’s Physicochemical and Mechanical Properties
Source: Polymers (Basel). 2020 Aug 6;12(8):1760. doi: 10.3390/polym12081760 (PMC7464250; doi:10.3390/polym12081760)
Supplement: Supplementary file 1 [file polymers-12-01760-s001.pdf]

Supplementary Materials

Table S1. Comparison of DS (HA-PDPH) based on activation of HA *via* EDC and DMTMM in dependence on the reaction time.

Reaction time	DS (PDPH-coupling *via* EDC) [a]	DS (PDPH-coupling via DMTMM) [a]
2 h	25%	4%
4 h	27%	7%
6 h	26%	9%
8 h	26%	12%
24 h	26%	28%
120 h	27%	40%

[a] 0.5 Eq of PDPH are related to the number of moles of HA.

Table S2. Summary of molecular weights (M_w) and corresponding hydrodynamic radii (R_h) of FITC-dextran in PBS applied to analyze the permeability of HA-microgels.

M_w (FITC-dextran)	R_h (FITC-dextran)
4 kDa	1.3 nm [66]
40 kDa	4.5 nm [66]
150 kDa	8.3 nm [66]
250 kDa	11.5 nm [65]
500 kDa	15.9 nm [65]
2,000 kDa	26.9 nm [65]

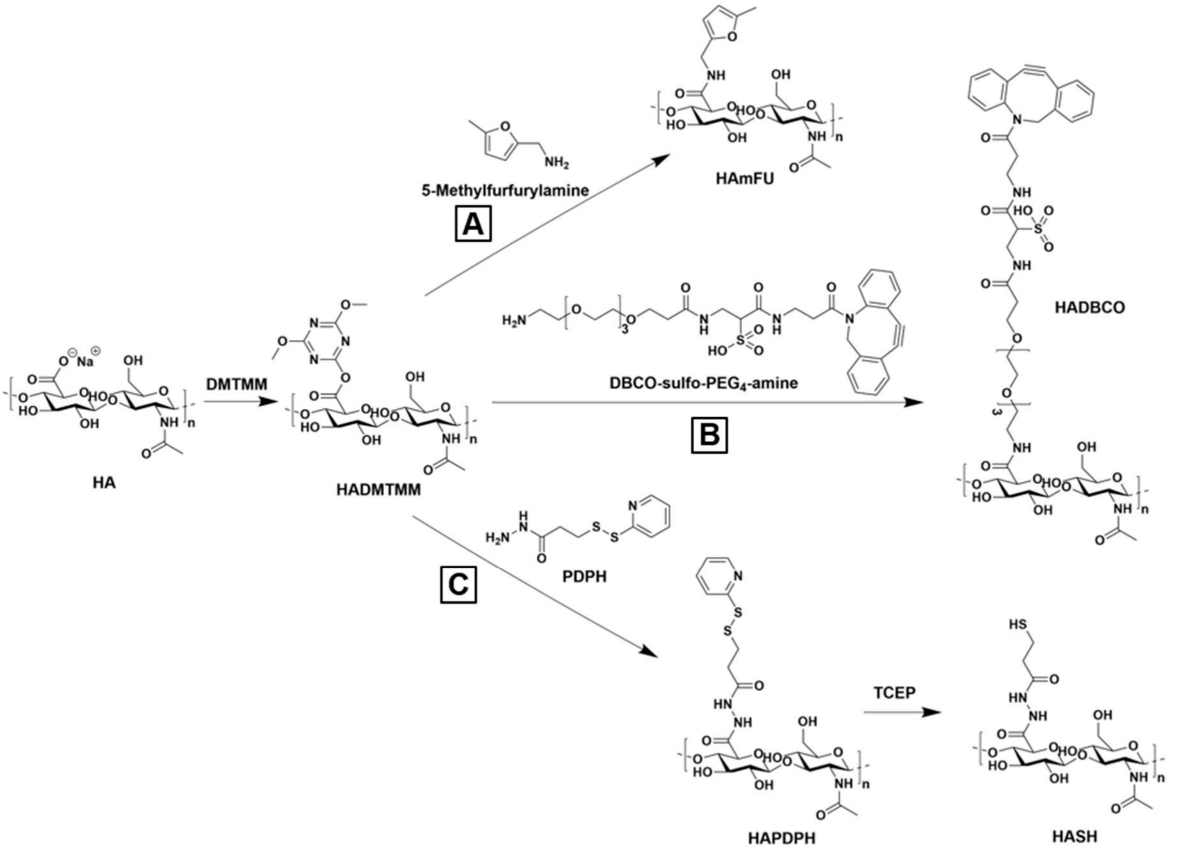

Figure S1. Synthesis of HA-derivates. Upon activation of HA-carboxylates *via* DMTMM, (**A**) 5-methylfurfurylamine is added to prepare HAmFU, (**B**) DBCO-sulfo-PEG$_4$-amine is coupled to yield HADBCO, and (**C**) PDPH is added, followed by treatment with TCEP, to obtain HASH.

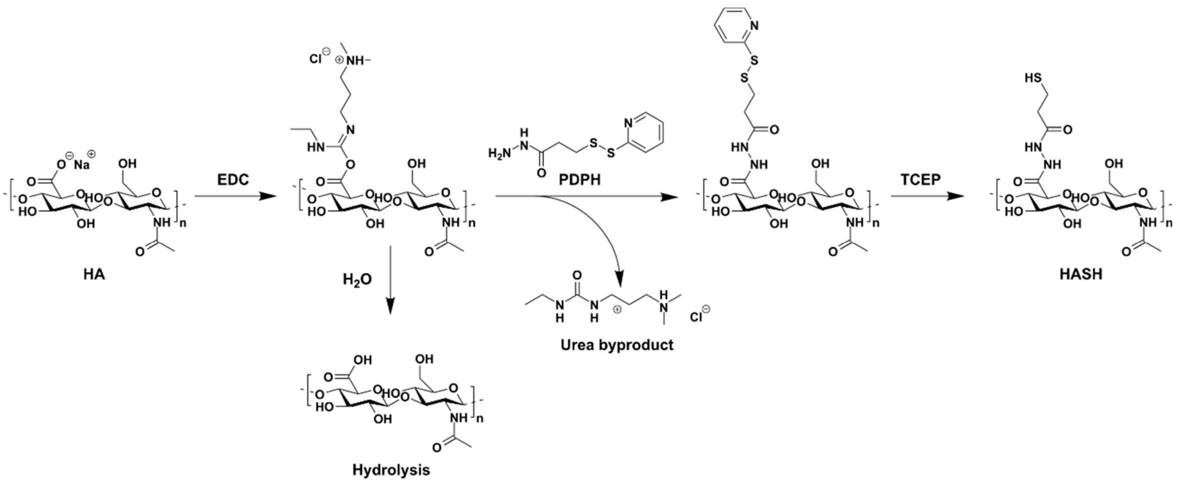

Figure S2. Synthesis of HASH employing EDC-activation chemistry. Upon activation, PDPH is coupled to the HA-backbone, followed by treatment with TCEP to cleave the internal disulfide group and yield HASH.

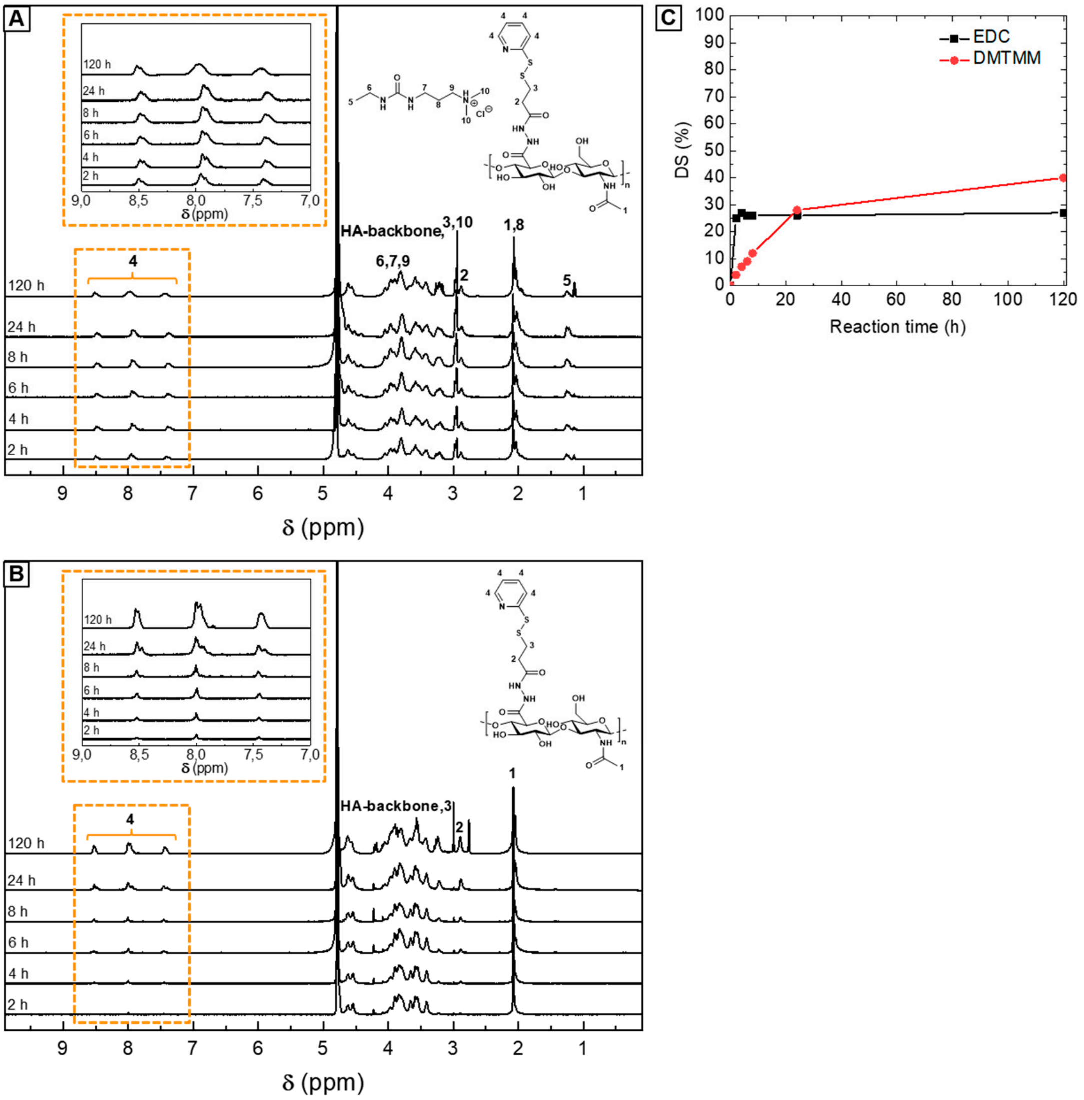

Figure S3. Systematic comparison of activation of HA-carboxyl groups by EDC and DMTMM depending on the reaction time yielding HA-PDPH. (**A**) ^{1}H-NMR spectra in D_2O of HA-PDPH synthesis utilizing EDC. After extensive dialysis for 96 h, urea byproducts still remain bound to polyanionic HA. To calculate the degree of substitution, signal 1 is compared to the aromatic protons 4, whereas the methylene signal 8 is roughly subtracted from 1. (**B**) ^{1}H-NMR spectra in D_2O of HA-PDPH synthesis in the presence of DMTMM. To determine DS, signal 1 is related to signal 4. Due to its insolubility in pure D_2O, caused by the high content of aromatic PDPH-moieties, HA-PDPH (DMTMM, 120 h) is measured in a D_2O-DMSO mixture (4:1). (**C**) Corresponding plot of DS depending on the reaction time and choice of activation reagent.

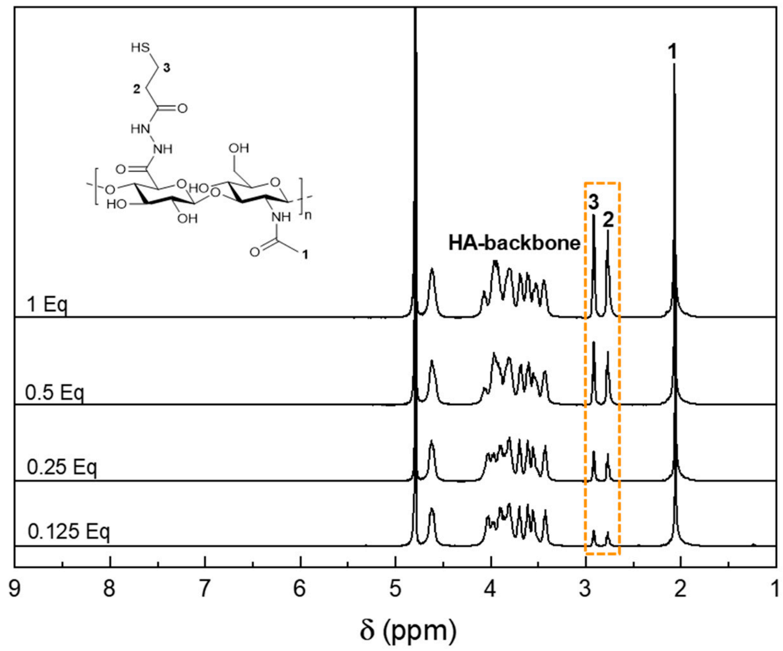

Figure S4. ^{1}H-NMR spectra of HASH in D_2O. The DS varies depending on inserted amounts of PDPH.

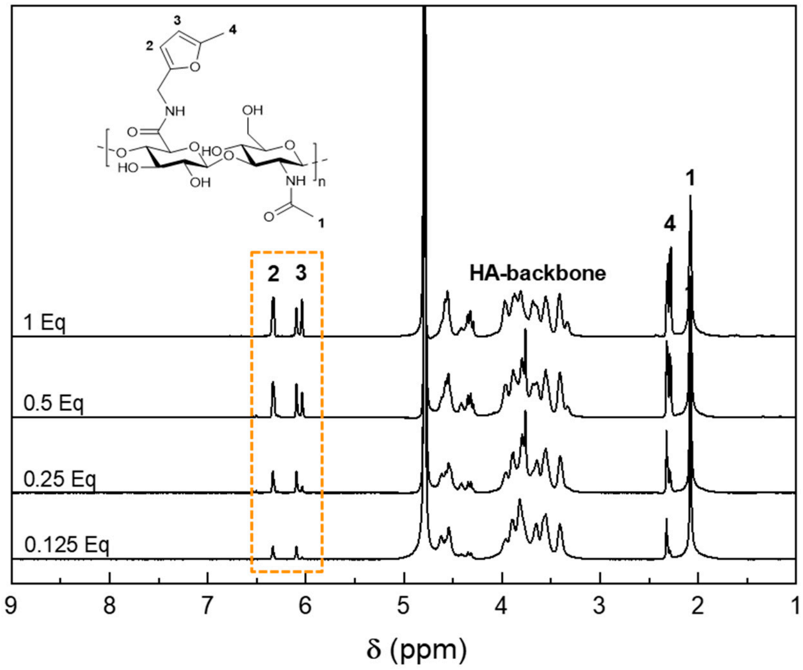

Figure S5. ^{1}H-NMR spectra of HAmFU in D_2O. DS is varied by the amount of 5-methylfurfurylamine added to the reaction mixture.

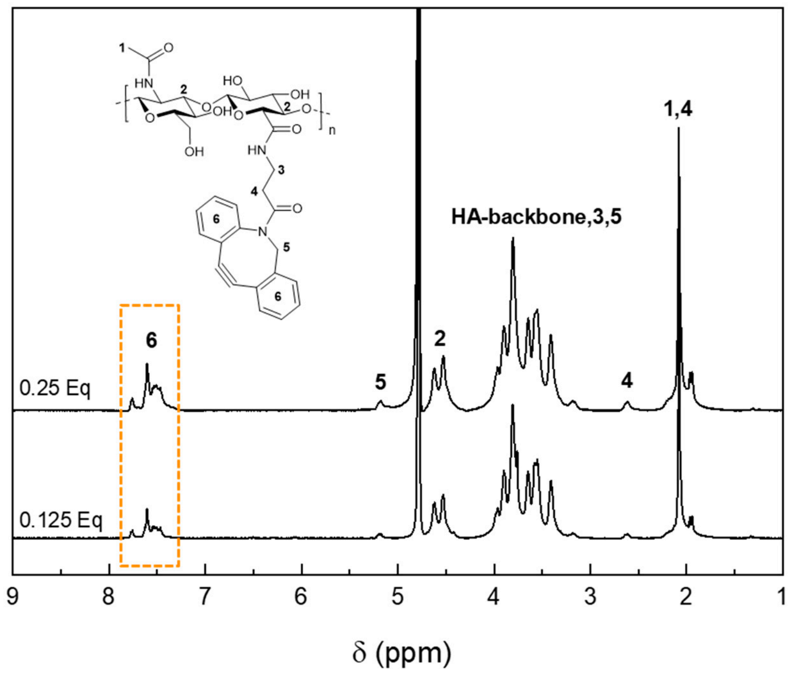

Figure S6. ^{1}H-NMR spectra of HADBCO in D_2O. DS is varied by the amount of DBCO-amine added to the reaction mixture.

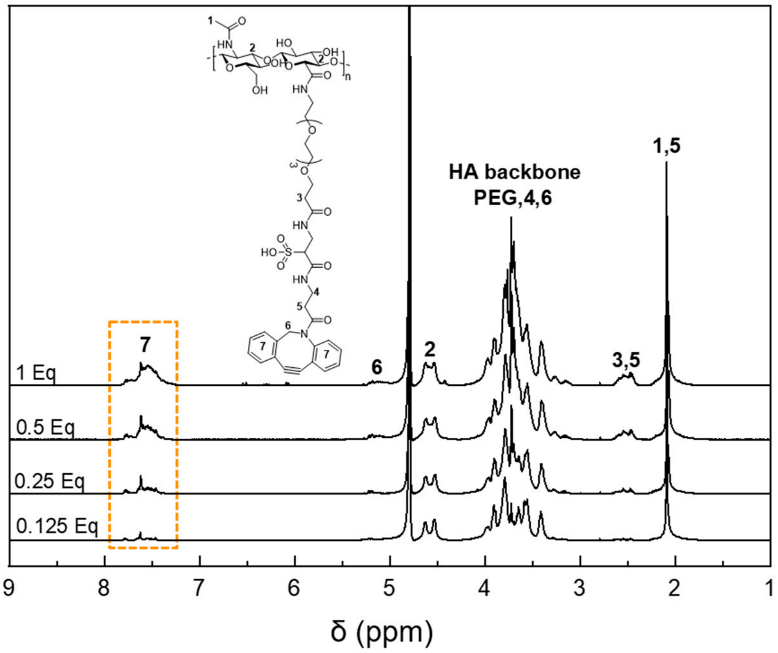

Figure S7. ^{1}H-NMR spectra of HADBCO in D_2O. DS is varied by the amount DBCO-sulfo-PEG_4-amine added to the reaction mixture.

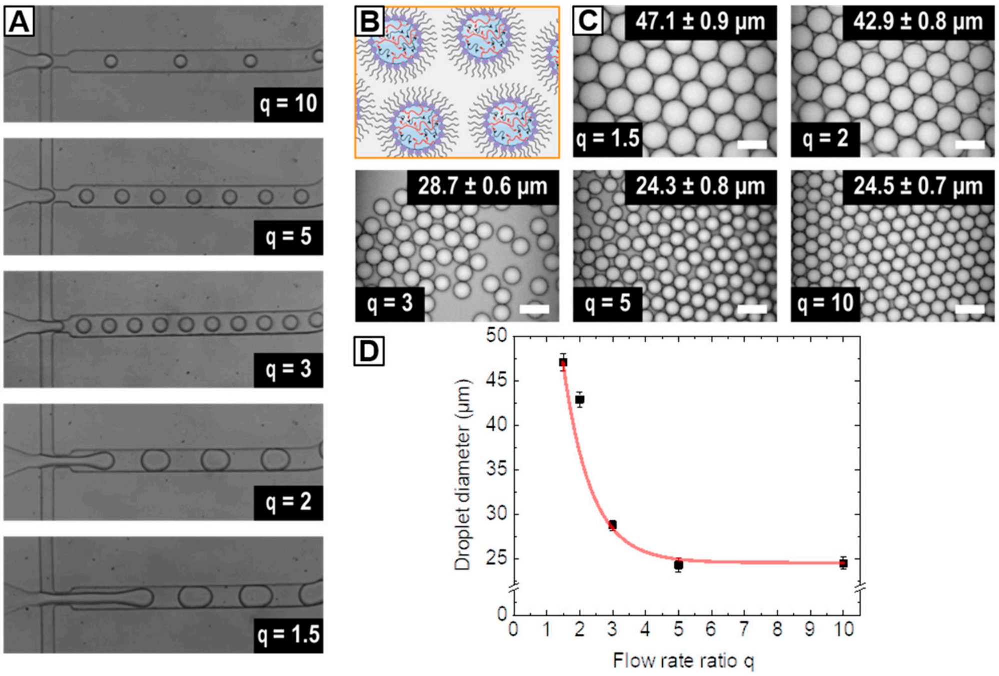

Figure S8. Microfluidic preparation of emulsion droplet populations by variation of the flow rate ratio *q* of oil with surfactant (continuous phase) and HA-derivate/PEG-crosslinker (dispersed phase). (**A**) Droplet formation depending on *q*, which is defined as the ratio of Q_c to Q_d. The droplet regime is switching from a dripping to a jetting regime with decreasing values of *q*. (**B**) Schematic of emulsified precursor droplets, which are stabilized in a fluorinated oil phase by a homemade triblock copolymer surfactant (Krytox®-Jeffamine®-Krytox®). (**C**) Brightfield microscopy images of resulting W/O emulsion populations. The scale bars for all images denote 50 µm. (**D**) Plot of flow rate ratio versus average droplet diameter (n = 100; mean ± SD).

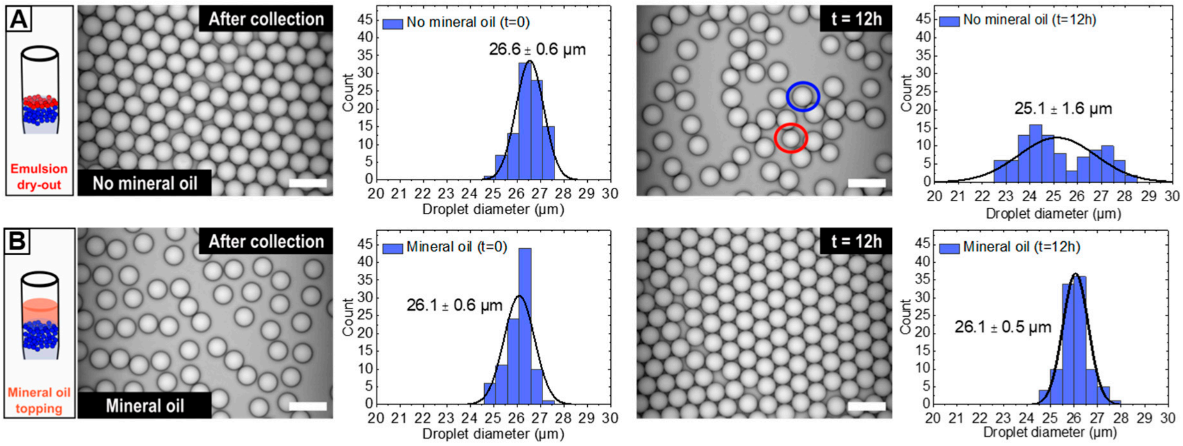

Figure S9. Brightfield microscopy images and corresponding droplet size distributions comparing batches prepared (**A**) without and (**B**) with mineral on top of the collected W/O emulsion. Left: Uniform emulsion droplets imaged directly after collection. Top right: Due to partial drying of emulsion droplets 12 h after gelation, a shift of particle diameter towards smaller sizes is observed. Bottom right: In contrast, droplets, which are protected by a topping of light mineral oil retain their uniform size. The scale bars denote 50 µm (n = 100; mean ± SD).

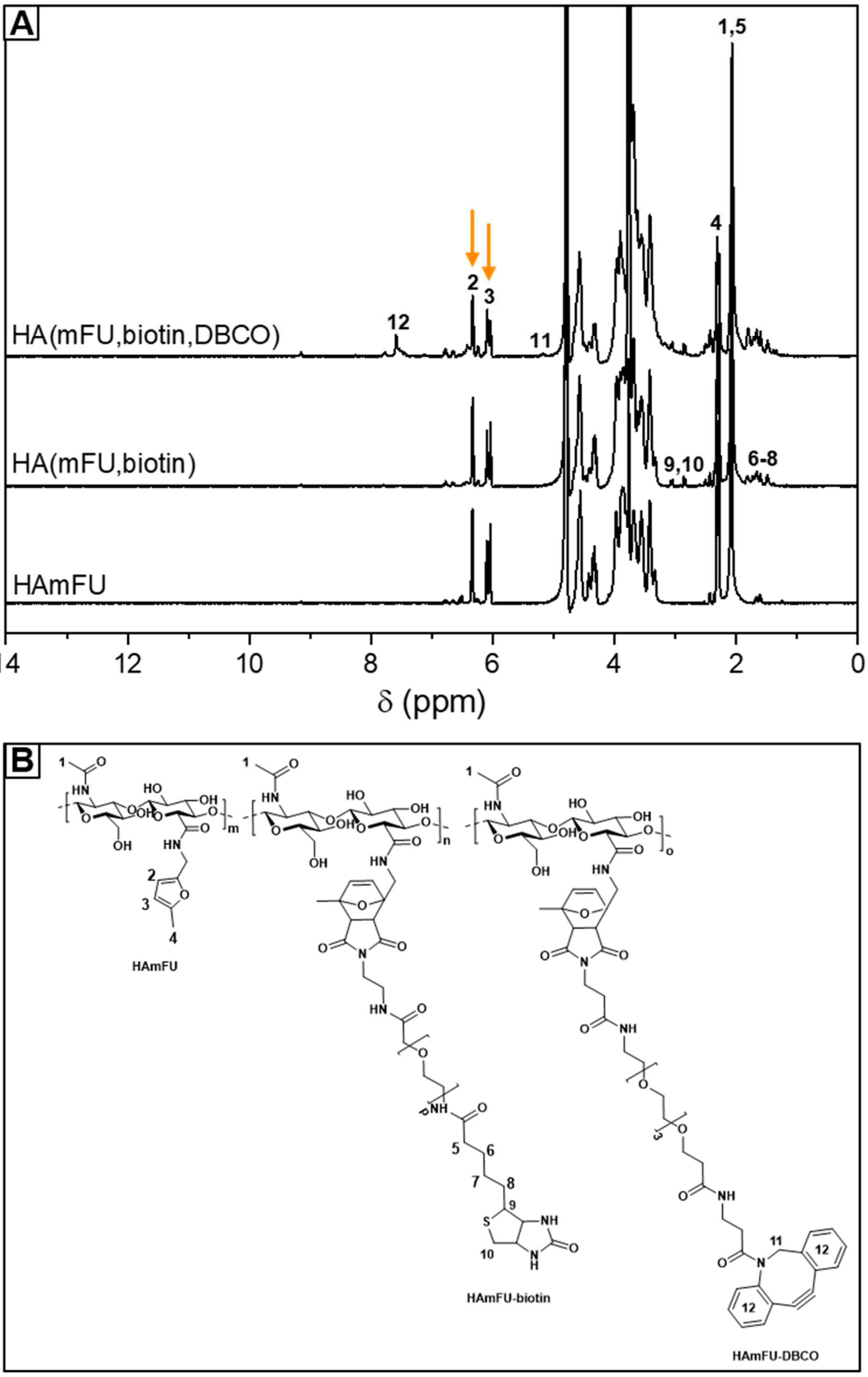

Figure S10. (**A**) ^{1}H-NMR spectra of HAmFU, HA(mFU, biotin), and HA(mFU, biotin, DBCO) in D_2O. The DS of biotin- and DBCO-substitution are calculated by comparing the signals (2,3) of unreacted methylfuran protons to the peaks of modified HAmFU. (**B**) Corresponding chemical structure of hetero-trifunctional HA.

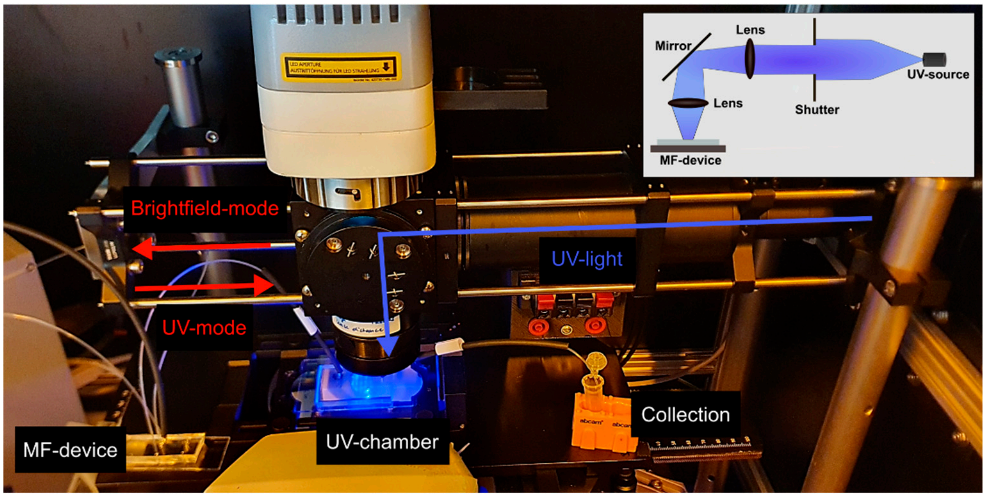

Figure S11. Homemade experimental UV-microscopy setup for on-chip preparation of HASH-microgels. By connecting a UV-source (OmniCure® S1500) to an inverted brightfield microscope, which is additionally connected to a high-speed camera (Phantom Micro eX4), prepared W/O emulsions are UV-polymerized *in situ* (250 – 450 nm, 44.6 mW cm^{-2}) within a microfluidic on-chip UV-chamber with a uniform height of 50 µm. Top right: Schematic illustration of the optical pathway of the UV-light onto the microscope stage and microfluidic device, respectively.
